# Incorporating qualitative research methods into the monitoring and evaluation of neglected tropical disease programmes: a scoping literature review

**DOI:** 10.1093/inthealth/ihab059

**Published:** 2021-10-06

**Authors:** Margaret C Baker, Kevin Bardosh, Elizabeth Fitch, Pamela S Mbabazi, Upendo Mwingira, Abdel Direny, Laura Dean, Elizabeth G Sutherland, Alison Krentel

**Affiliations:** Global Health Division, Research Triangle Institute (RTI) International, Washington DC, WA 20005, USA; Center for One Health Research, School of Public Health, University of Washington, Seattle, WA 98195, USA; Global Health Division, Research Triangle Institute (RTI) International, Washington DC, WA 20005, USA; Department of Neglected Tropical Diseases, World Health Organization, 1221 Geneva 27, Switzerland; Global Health Division, Research Triangle Institute (RTI) International, Washington DC, WA 20005, USA; CORUS International, Washington DC, WA 20036, USA; Department of International Public Health, Liverpool School of Tropical Medicine, Pembroke Place, Liverpool, L3 5QA, UK; Global Health Division, Research Triangle Institute (RTI) International, Washington DC, WA 20005, USA; School of Epidemiology and Public Health, University of Ottawa, K1G5Z3, Canada; Bruyere Research Institute, Ottawa, K1N 5C8, Canada

**Keywords:** disease control, mass drug administration, neglected tropical diseases, participatory methods, qualitative research, programme design

## Abstract

This publication addresses the limited use of qualitative methods in neglected tropical disease (NTD) programmes. It describes a scoping literature review conducted to inform the development of a guide to inform the use of rapid qualitative assessments to strengthen NTD mass drug administration (MDA) programmes. The review assessed how qualitative methods are currently used by NTD programmes and identified qualitative approaches from other health and development programmes with the potential to strengthen the design of MDA interventions. Systematic review articles were reviewed and searched using key terms conducted on Google Scholar and PubMed. Results show that methods used by NTD programmes rely heavily on focus group discussions and in-depth interviews, often with time-consuming analysis and limited information on how results are applied. Results from other fields offered insight into a wider range of methods, including participatory approaches, and on how to increase programmatic uptake of findings. Recommendations on how to apply these findings to NTD control are made. The topic of human resources for qualitative investigations is explored and a guide to improve MDAs using qualitative methods is introduced. This guide has direct applicability across the spectrum of NTDs as well as other public health programmes.

## Introduction

The medically and epidemiologically diverse group of 20 neglected tropical diseases (NTDs) prioritised by the WHO affect more than 1 billion people globally. More than 200 000 people die each year from snakebites, rabies and dengue, and hundreds of millions of others have experienced severe disability, disfigurement, stigmatisation and discrimination due to the full range of NTDs.^1,2^ Because NTDs affect people living in the most marginalised conditions, progress reducing the burden of NTDs is seen as a proxy indicator for measuring progress towards sustainable development goals and universal health coverage with equitable access to health services, leaving no one behind.^2,3^

The WHO's 2021–2030 road map for NTDs focuses on increased country leadership and integration, as well as achieving disease-specific impact.^2^ The roadmap describes intervention strategies by disease, including preventative chemotherapy (PC) as a key intervention for 10 of the 20 NTDs. PC is the large-scale administration of medicine, often delivered through mass drug administration (MDA) to treat people in endemic areas, regardless of their infection status. For the fifth consecutive year, the global NTD community has delivered more than 1 billion treatments annually since 2015.^4^ Delivery on this scale has resulted in lymphatic filariasis and trachoma elimination as a public health problem in 17 and 11 countries, respectively, and onchocerciasis transmission has been eliminated in four countries in the region of the Americas.^2^ The size of this achievement is a testament to what can be achieved when partners (including endemic country governments, the United Nations, donors, pharmaceutical company donation programmes, researchers and non-governmental organisations [NGOs]) work together.

As we turn our attention to reaching programme targets in ‘the last mile’, social sciences and qualitative methods could make a difference. For example, a programme may find ongoing low coverage at district or subdistrict level. Managers may also find that transmission continues at higher-than-expected levels, despite multiple rounds of MDA.^5^ As they face these issues, programme managers are increasingly grappling with challenging questions: Who are we not reaching? Why? What is their personal risk? Are they contributing to ongoing transmission? What do we need to do differently?

While a strong suite of quantitative tools has been developed in recent years to support the monitoring and evaluation of PC NTD programmes, these do not provide full answers to all the questions posed above.^6^ For example, a coverage survey may identify that 20% of persons surveyed have a fear of MDA drugs, but understanding what they fear and why requires a deeper contextualised understanding. The number and/or size of tablets, the result of rumours, lack of trust in the people or organisation delivering treatment, the fear of side effects experienced in the past, a mix of all these reasons or something else altogether? And what can programme managers do to help people overcome these concerns? Who should deliver the message of drug safety and how? Concern has also been voiced that quantitative surveys may be missing the same people missed during MDA, and so they remain hidden to the programme, both during MDA and during the assessments.^7^

Qualitative tools can be used to give a stronger voice to the communities and health workers who are often best placed to identify and solve programmatic challenges, and leads to the creation of more person-centred approaches to NTD programme delivery. The NTD 2020–2030 road map is complemented by other documents that provide frameworks for thinking more in depth about sustainability, investment, monitoring and evaluation.^8,9^ The monitoring and evaluation document stresses the need for increased use of qualitative methods, thus addressing the limited use of social science methods in NTDs that has been called out in several publications.^4,10–14^

To address this gap, we conducted a rapid scoping literature review designed to inform the development of an off the shelf ready guide on the use of qualitative methods in NTD programmes, with a focus on MDA programmes. This paper presents the results of that review and introduces the guide. While drawing mainly from the experience of PC NTDs, this guide also has direct applicability across the spectrum of NTDs, as well as other public health programmes.

## Methods

We conducted this scoping literature review with the aim of gaining a better understanding of how qualitative methods are currently being used by PC NTD programmes on one hand and, on the other, to identify a range of qualitative methods being used in other public health programmes that could drive innovation within the NTD space. We therefore conducted two separate reviews: one focused on PC NTD literature and another on other health and development fields. As methods were identified we also sought to understand how their application was used to inform programme design.

The two reviews were carried out by two different researchers (AK and KB) and the results were regularly discussed with the project team (MCB, EF, AK and KB). The review on the use of qualitative methods used in PC NTDs focused on searches of specific known qualitative methods within the PC NTDs. The initial list of methods was derived from a breakout session at the 2019 Coalition of Operational Research for NTDs meeting in National Harbour. During that breakout session, researchers and programme managers identified a list of methods previously used in NTD programmatic research. This list formed the basis for searches within PubMed, combining the method with the disease name. From there, additional papers were identified through the snowball technique. To be included in the final list, papers needed to provide sufficient information on what triggered the use of the method; a summary of the data collection methods; sufficient results to comment on quality and how steps were taken to integrate findings into the programme; and (if possible) comments about the time taken for the research study, human resource needs and costs to give an idea of the feasibility of its use. Where details about resources required were not available, the researcher (AK) estimated the time and human resources for the methods based on past research experience. The final list included a selection of example papers that represent use of the different methods captured during the scoping review. The review of the broader health and development literature started with 15 systematic reviews on qualitative research from the fields of childhood vaccinology, Ebola, health emergencies, malaria, healthcare delivery, randomised controlled trials (RCTs), substance abuse and TB. From these, five rapid qualitative toolkits were identified and reviewed. In addition, searches were performed on Google Scholar and PubMed using a wide variety of search terms since the goal was to find a wide range of non-NTD literature. This included starting with broad terms such as ‘infectious disease’, ‘health’, ‘development’ AND ‘qualitative research’, ‘qualitative methods’ ‘ethnography’, ‘participatory research’ AND ‘systematic review’ and ‘review’, then narrowing the focus to those that would lend themselves to informing the design of MDA programmes for PC NTDs.

In both reviews, once papers were identified, the full paper was read and a standardised framework in Excel (Microsoft Corporation, 2018. Microsoft Excel. Retrieved from https://office.microsoft.com/excel) was used to systematically collect information on why the method was selected, the reported feasibility of adaptation for routine programmatic use in terms of resources (human, financial and time) and the reported uptake of results.

All the authors reviewed the results and discussed how these should be applied to the design and content of the new Guide to Improving MDA Using Qualitative Methods (https://www.ntdtoolbox.org/toolbox-search/guide-improving-mda-using-qualitative-methods). In doing so, the diversity of authors’ perspectives was leveraged, including experiences as programme managers, WHO NTD staff, implementing partners, monitoring, evaluation and learning advisors, as well as researchers. The guide development was further informed by EF and MCB’s engagement with their colleagues (at country and headquarters level) on the Act to End NTDs East programme funded by United States Agency for International Development (USAID), which supports the implementation of NTD programmes in 12 countries.

## Results

### Qualitative methods used in NTD research

**Figure 1.**
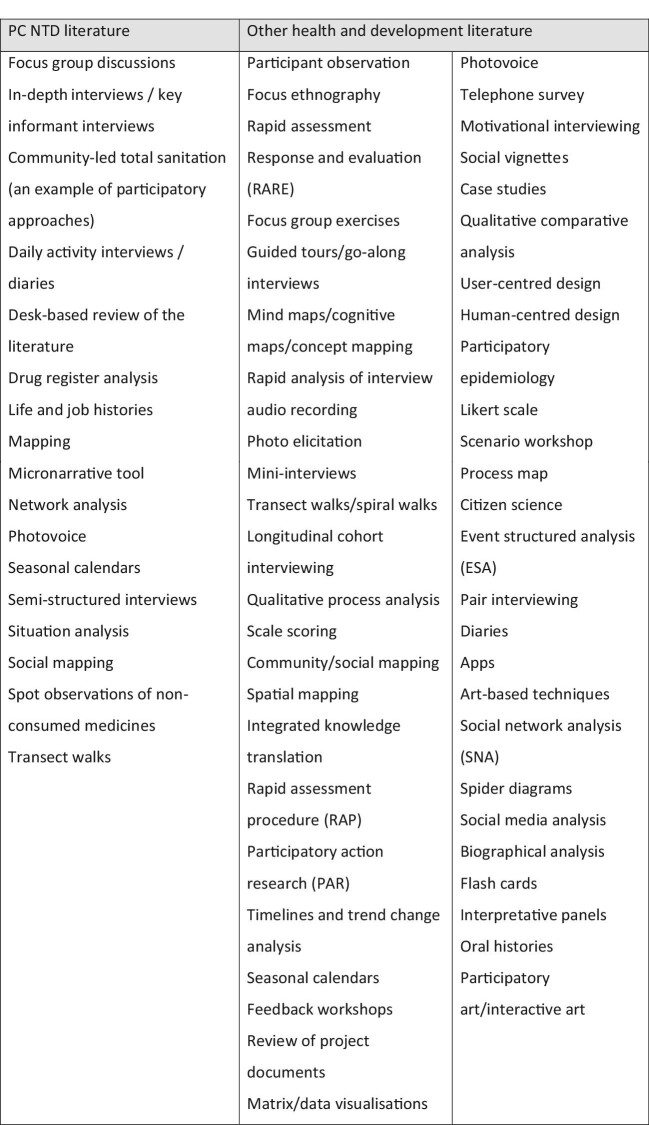
Summary of qualitative methods found in the literature search.

A total of 24 papers were identified for further analysis, consisting of 22 unique research papers and 2 review papers.^7,15–38^ Focus group discussions (FGDs) and in-depth interviews (or key informant interviews) were the most commonly used methods in the papers reviewed. There were examples of a range of other methods, including participatory approaches (Figure 1). For example, Fleming et al. used pictorial diaries, combined with in-depth interviews, to assess the opportunity costs for community drug distributors (CDDs) implementing MDAs.^31^ Most of the studies included in the review used more than one tool, with FGDs and in-depth interviews frequently used together, triangulating results across study types.

Study participants commonly included the following groups: males and females (often interviewed separately), community and opinion leaders, CDDs and frontline health workers. Additionally, other important groups of interest were sometimes identified as participants in FGDs and in-depth interviews. One study from India used in-depth interviews with those individuals who did not take medicines during the last MDA.^18^ Another study interviewed CDDs who had resigned from carrying out MDA activities, to understand their rationale for stopping.^29^ Only two studies reviewed included children or adolescents as informants in their studies.^15,37^

**Rationale for selection of qualitative method**: Most of the selected papers reported using qualitative studies to better understand MDA coverage results. Some sought to increase their understanding on perspectives of the role of the deliverer (e.g. CDD) in the MDA process.^14,17,19,27–29,31,35,37,38^ Some of these papers were looking to build on knowledge already generated by coverage surveys.^14^ As Brieger writes, ‘A coverage survey is not the ideal instrument to enquire about beliefs and attitudes, and thus a smaller survey may be needed to document beliefs and test their association with coverage’.^20^

**Team structure and logistics**: Most of the papers did not describe in detail the team structure, time taken or logistical requirements to implement the studies. The reviewer (AK) used her experience to make a rough estimate of the amount of effort (low, moderate, high) needed to collect and analyse the data. For example, FGDs and in-depth interviews typically reported using recordings and producing verbatim transcripts. This can be a time-consuming process with an average 1-h FGD requiring 4–6 h to transcribe and a 1-h in-depth interview requiring 2–4 h to transcribe. Most studies were deemed to have moderate to high human resource needs, particularly when the sample size was large.

**Knowledge translation:** Some of the study results included the need for more in-depth knowledge and perceptions of the MDA; reasons for not wanting to take the treatment offered; an understanding of which groups of people missed treatment; MDA delivery barriers and enablers; rich descriptions on the challenges to reach specific communities (e.g. geographic barriers, higher social economic status [SES]); and CDD-related challenges (e.g. poor numerical literacy and high opportunity costs).^14,15,17–21,23,24,27,29,31,33–35,37,38^

**Evaluation of research impact:** While most of the reviewed studies included a list of programme recommendations, only two papers provided information on how (or if) their findings were used by the NTD programme.^32,35^ These descriptions included documentation of programme changes based on the findings and future MDA coverage in areas where changes were implemented.

Related to this, a systematic review article highlighted that the types of methods used make it difficult to conclusively attribute any change in MDA coverage to changes made by the programme.^26^ Some authors also commented on the inability to generalise qualitative results beyond the context. Ames et al. highlighted the fact that a context description was often missing, as was any reflection on the role that the researchers may have played in influencing study results.^16,28,29^

### Qualitative methods used in the broader health and development field

A total of 14 systematic review papers were identified for further analysis from the fields of vaccinology, Ebola, health emergencies, malaria, healthcare delivery, RCTs, substance abuse and TB.^39–52^ From these, 25 articles were selected for full review based on their relevance to the aims of the review.^52–76^ Additionally, five rapid qualitative toolkits of relevance were identified through a more general internet search.^77–80^ A total of 52 different qualitative methods were identified (Figure 1). Many were participatory and team-based in nature and in line with ‘implementation science’ approaches.

**Rationale for selection of qualitative method**: The selected articles provided a broad range of reasons for the use of qualitative methods. These included the need to generate formative insights to influence the design of programmes, such as vaccinations, childhood feeding, Ebola and malaria control,^52–54,66,68,71,76^ to increase community and stakeholder participation, including with racial and ethnic minority communities in HIV/AIDS prevention,^70^ injecting drug users^73^ and those undergoing aortic valve implants,^56^ and to understand specific operational shortcomings or failures of accepted approaches and policies, many of which were comparative studies reflecting on implementation across multiple countries.^58,60^,^61,75^

**Team structure and logistics**: Almost all the studies reviewed used a senior, trained social scientist, either directly involved in data collection and analysis or in training and supervision. A range of different types of personnel were employed for data collection, including nationally trained social scientists and community engagement staff (not trained in social science).^58,74^

Many studies were often part of longer term social science engagement performed over a few years, which served to also build capacity in research and knowledge translation.

**Knowledge translation:** Studies described a process of knowledge translation that started from the beginning, with a built-in process for co-design with different stakeholders and a review of existing programme materials. Stakeholder engagement continued throughout the study and resulted in adapting research questions and methods in response to preliminary results and changing programme needs. Towards the end, stakeholders analysed the relevance of the results. Methods used to facilitate this partnership, and to build the trust and respect needed for it to be effective, included member checking, peer debriefing, group analysis and stakeholder workshops (including community working groups and community advisory boards).^60,67,70,73,69,75^ Some studies used voting/ranking methods with end users (e.g. programme staff) to explore the feasibility of recommendations. Many papers stressed that rapid assessments can often be integrated with existing programmes to reduce costs and that they should be integrated with attention to the programme cycle.^56,60,61,73^

**Evaluation of research impact:** Surprisingly, few studies evaluated the impact of research findings on programme implementation, although some argued that integrating an evaluation component into a rapid qualitative tool is very important.^69,75^

## Discussion

This scoping literature review showed that qualitative methods are used by PC NTD programmes for a variety of reasons, including to better understand access to treatment. However, most studies restricted their methods to in-depth or key informant interviews and FGDs, and many reported the use of time-consuming analysis methods. Few reported on how results were taken up by programmes or evaluated the impact of using results on the problem that the study was designed to address. This confirmed the authors’ observations that there is a need to better align the use of qualitative methods within NTD programmes, to provide more timely information and to tailor them towards problem solving. An increased use of qualitative methods can also be used to allow the voices of the community and beneficiaries to be heard more clearly. These perspectives—which include women, children, the rural and urban poor and people with disabilities, some of which may be caused by NTDs—are often missing in the world of NTD programming and policy, yet are vital if we are to achieve equity and reach programme goals.

Our review of the use of qualitative methods in other areas of public health and development found a much wider range of methods in use (including several rapid participatory approaches), and we found several studies that paid a lot more attention to knowledge translation and programmatic uptake of findings. However, even here, there was a surprising lack of studies that evaluated the impact of using results on programme outcomes.

**Figure 2.**
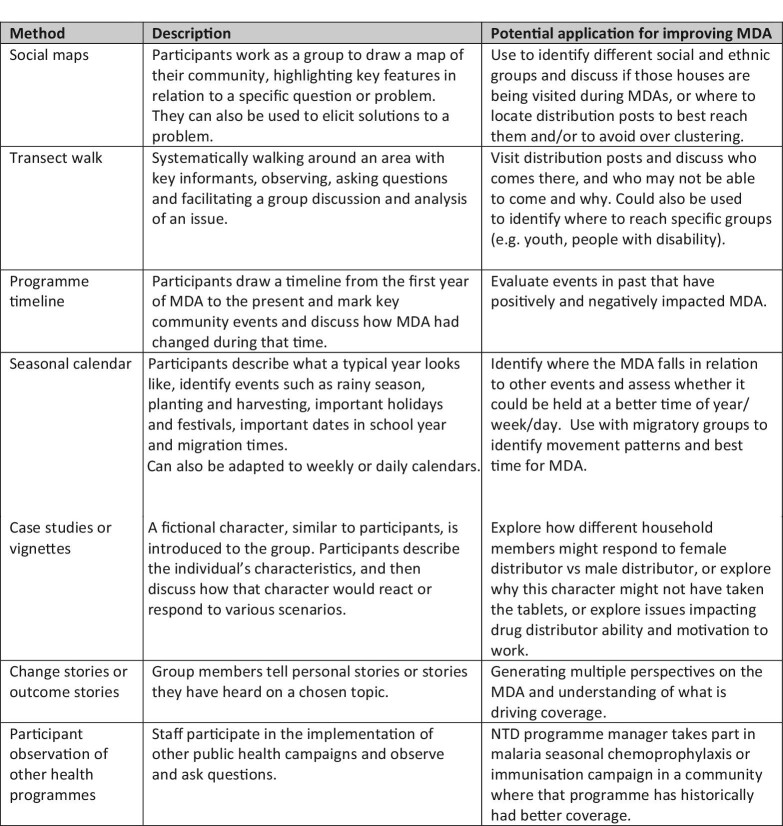
Summary of qualitative and participatory methods, in addition to focus groups and in-depth interviews, adopted for use in the Guide to Improving MDA using Qualitative Methods.

Of the methods identified in both literature reviews (Figure 1), we recommend several methods that could be adapted and used to inform and improve future design of MDAs. These are summarized in Figure 2, with examples of potential applications to NTD programmes provided. This is not intended to be an exhaustive list of possible methods (which would be potentially overwhelming), nor does it claim to be a ‘best list’, but offers a list of methods that are quick to implement, require minimal expertise in qualitative and or participatory research, and can easily be applied to solving PC NTD programmatic challenges.

If results generated by qualitative studies are to be applied to specific programme challenges, knowledge translation and uptake is needed and attempts to implement changes then need to be evaluated. This requires the research team (be they researchers or programme monitoring and evaluation [M&E] staff) and the implementation team working together at every stage.^11,60,67,69,70,73,75^ At the beginning, they need to work together to clearly define the problem and research questions. NTD programmes have data from several sources that can be used to inform this step, including coverage surveys, routinely collected subdistrict level coverage data (broken down by age and/or gender), supervisor and other programme reports, records of post-MDA meetings, impact survey reports and tacit knowledge gained by staff from implementation experience. Collaboration needs to continue during data collection with a willingness to adapt instruments and methods. The relatively short timeframe available for evaluation between rounds of MDA further underscores the need for evaluators and implementers to collaborate closely for successful data collection and data use.

To ensure successful programme learning and adaptation, study results need to be jointly reviewed by researcher and implementer and a set of feasible recommendations co-designed. The nature of programme implementation is that it will never be perfect, especially in the settings where NTD programmes operate, the scale at which they operate and with the resources available to them. It is therefore easy to detail a long list of things that could be improved, be they training, supervision, data collection, social mobilisation, attitudes or staffing. The key is to identify the, usually considerably smaller, list of things that are most likely to affect the desired change. This is as much an art as a science. Of the methods identified in this study that facilitate that process, one that we particularly liked was the Risk, Attitude, Norms, Ability and Self-regulation (RANAS) model of behavioural change commonly used in water and sanitation. With the RANAS methodology, a framework is provided for programme implementers to use their survey results to adapt and design appropriate messages that respond to the gaps identified in the assessment.^81^

We found a significant lack of studies evaluating the impact of programmatic changes made based on study results. When evaluations are conducted, they usually use more quantitative methods and quasi-experimental designs like ‘before and after’ studies, with or without comparison areas. However, there are now also newer qualitative evaluation methods available, which take into consideration the complexity that ensues from the interaction of multiple programmatic changes that interact with the people and their environment in multiple, and sometimes unexpected, ways. These so-called ‘complexity aware’ methods include techniques such as most significant change, ripple effects mapping and outcome harvesting.^82,83^

The question of who is best suited to undertake qualitative research in the context of a programme is often hotly debated: trained researchers, M&E staff or programme managers? We have used the diversity of experience of our authors (including both programme implementers and researchers) to consider here the advantages and disadvantages of these options. NTD programmes often work with researchers trained in qualitative methods. These may be found in other units within the ministry of health, in other government sectors, in local universities and other research organisations, or with their international partners. Trained researchers have more experience with design, data collection and methods, which can lead to more efficiency and quality. They are less biased by the programme beliefs on what is happening and why, although of course they are not immune to their own biases and they lack knowledge on programme context. They usually have more time to devote to, and a preference for, more sophisticated study design: this can be a double-edged sword when time is of the essence. Engaging with outside experts requires additional funding allocated to evaluation in the programme budget and has at times produced results that are not aligned with the programme planning and budgeting cycles during which uptake of recommendations can be made. Conversely, qualitative research is often carried out by NTD programme implementers, including national or regional NTD programme managers and their teams (including M&E staff), district level health teams and international implementing partners. Programme staff have a better understanding of programme context and may have a more intuitive sense of where to probe in interviews. They tend to have more contextual knowledge that can facilitate interpretation of results, quickly filtering through all the noise. However, they may lack training in seeking out counter hypotheses, and may stop probing quickly if initial answers are in line with what they already believe. They are well positioned to apply results, although they are not immune to getting lost in lists of recommendations. As their time is already accounted for in programme budgets, they do not require additional funding, although there are opportunity costs to consider, especially when efficiencies are lost during study planning, data collection and analysis. The final decision on who performs the study will differ depending on context, funding and staff availability, and perhaps ideally will include a mixed team of both researchers and programme staff.

Regardless of who conducts the studies, the need for capacity strengthening on qualitative research for NTD control and elimination has been clearly stated.^8–12^ This need has begun to be reflected in the global NTD community through inclusion of a chapter on qualitative methods in the NTD Roadmap M&E framework and the launch of a new community of practice of researchers from different social science backgrounds focused on NTDs called iCHORDS (Improving Community Health Outcomes through Research, Dialogue and Systems Strengthening).

To respond to the need for capacity strengthening on qualitative methods, we used the results from this study, combined with a human-centred design approach^84^ to develop A Guide to Improving MDA using Qualitative Methods. We used rapid literature to help frame subsequent discussions with researchers, technical experts and NTD programme implementers to define the problem or issues that needed to be addressed, brainstormed how to address those problems and jointly developed a prototype guide. We also built on a previous manual developed by the WHO^10^ that drew on experiences from other health programmes but was reportedly difficult to use due to its length, complexity in language and requirement for more resources than are usually available.

The Guide to Improving MDA using Qualitative Methods (https://www.ntdtoolbox.org/toolbox-search/guide-improving-mda-using-qualitative-methods) provides practical guidance on topics like budgeting, includes templates for training agendas and data analysis, has modifiable questionnaires and avoids overly scientific language. It requires the study team to work with the programme team responsible for designing the MDA intervention through six steps: scoping to define the research question, methods selection, data collection, data analysis, programme adaptation and programme evaluation.

The limitations of this study include the fact that we did not scope grey literature due to time constraints, including NGO project documents, which may have highlighted the use of additional qualitative methods and provided a richer description of the uptake of results. There would be value in a future study that looks at these sources. The aim of the literature review was to provide a snapshot of some of the range of qualitative methods that have been used in NTD and public health programmatic research. As such, it does not claim to be exhaustive, however, it was useful in framing the prototype and we believe it will help drive current discussions on the role of qualitative methods in NTDs. Our focus on MDA, drawn mostly from experiences with PC NTDs, is also a limitation. Future work should address the use of qualitative research for other elements of NTDs, including health system strengthening and other types of intervention such as disease management, disability and inclusion. Finally, the guide described also needs to be tested and evaluated.

## Supplementary Material

ihab059_Supplemental_Files
